# Rational quinidine dosage regimen for atrial fibrillation in Thoroughbred racehorses based on population pharmacokinetics

**DOI:** 10.3389/fvets.2024.1454342

**Published:** 2024-10-07

**Authors:** Taisuke Kuroda, Yohei Minamijima, Christopher Ken Kinman, Yuji Takahashi, Yusaku Ebisuda, Kaori Inoue, Hiroshi Ishikawa, Hiroshi Mita, Norihisa Tamura, Toshio Nukada, Pierre-Louis Toutain, Minoru Ohta

**Affiliations:** ^1^Clinical Veterinary Medicine Division, Equine Research Institute, Japan Racing Association, Shimotsuke, Japan; ^2^Drug Analysis Department, Laboratory of Racing Chemistry, Utsunomiya, Japan; ^3^Sports Science Division, Equine Research Institute, Japan Racing Association, Shimotsuke, Japan; ^4^Ritto-Training Center Racehorse Hospital, Japan Racing Association, Ritto, Japan; ^5^Comparative Biomedical Sciences, The Royal Veterinary College, London, United Kingdom; ^6^INTHERES, Université de Toulouse, INRAE, ENVT, Toulouse, France

**Keywords:** quinidine, atrial fibrillation, horse, population pharmacokinetics, dosage regimen

## Abstract

**Introduction:**

Quinidine (QND) sulfate is an effective treatment for atrial fibrillation (AF) in horses, and several dosage regimens have been proposed to address its wide variability in response and potential adverse effects. The purpose of this study was to analyze the variability in plasma quinidine concentrations using population pharmacokinetics to determine an effective and safe dosage regimen for Thoroughbred horses.

**Methods:**

Six healthy Thoroughbred horses were treated with 20 mg/kg quinidine sulfate dihydrate (16.58 mg/kg QND base) administered PO or 5 mg/kg quinidine hydrochloride monohydrate (4.28 mg/kg QND base) administered IV (single administration), and blood samples were taken regularly. Four healthy horses were treated with 20 mg/kg quinidine sulfate dihydrate administered twice (every 6 h) via PO route. For the other 19 Thoroughbred racehorses that developed AF, blood samples were taken during quinidine therapy. Quinidine concentrations were measured in all plasma samples using liquid chromatography with tandem mass spectrometry, and the data from 29 horses were modeled using a nonlinear mixed-effects model, followed by Monte Carlo simulations (MCS).

**Results:**

The median quinidine concentration for successful sinus rhythm conversion was 2.0 μg/mL (range: 0.5–2.7 μg/mL) in AF horses, while a median concentration of 3.8 μg/mL (range: 1.6–5.1 μg/mL) showed adverse effects. MCS predicted that plasma quinidine concentrations for quinidine sulfate dihydrate PO administration (loading dose: 30 mg/kg, maintenance dose: 6.5 mg/kg q 2 h) reached 1.4, 2.0 and 2.7 μg/mL in 90, 50 and 10% of the horse populations, respectively. Increasing the loading dose to 45 mg/kg and the maintenance dose to 9 mg/kg q 2 h, the plasma concentrations achieved were 1.9, 2.8, and 3.8 μg/mL in 90, 50, and 10% of horse populations, respectively.

**Discussion:**

Using simulations, different empirical dosing regimens were proposed to achieve plasma quinidine concentrations immediately or progressively, representing a tradeoff between optimizing therapeutic effects and minimizing adverse effects. A combination of these dosing regimens is recommended to gradually increase the therapeutic concentration levels of quinidine for safe and effective treatment of AF in racehorses.

## 1 Introduction

Atrial fibrillation (AF) is a common performance-limiting arrhythmia in horses, and it is important to convert AF to normal sinus rhythm in order to recover racing performance (1–4). The incidence of post-race AF is between 0.03 and 0.3% (1, 2, 4–6). Several treatments have been proposed for AF, including quinidine (QND) (7), propafenone (8), flecainide (9, 10), amiodarone (11), and electrical cardioversion (12, 13). Among these, QND is the most popular pharmacological treatment for Thoroughbred horses (14, 15).

QND is a Vaughan–Williams class Ia antiarrhythmic agent that prolongs cellular action potential by blocking sodium channels, as well as potassium channels, resulting in the conversion of AF in horses into sinus rhythm (16, 17). Intravenous (IV) administration of quinidine gluconate or oral (PO) administration of QND sulfate has been reported in horses (7, 16), and various QND dosage regimens have been suggested for treatment of adult horses with AF (7, 18–21). Currently, QND sulfate is the only pharmacological agent available for oral administration because quinidine gluconate has been discontinued from the market. Oral QND treatment is difficult because it can cause serious side effects in horses, including death (16, 18); therefore, precise dosing recommendations are required to ensure both the success of the treatments and the minimum occurrence of adverse events.

A classic dosing regimen for horses without heart failure consists of QND sulfate PO (by nasogastric tube), 22 mg/kg q 2 h until: (i) conversion to sinus rhythm, (ii) occurrence of adverse or toxic effects, or (iii) administration of a total of 4 (or 6) doses. In addition, the plasma quinidine concentration should be measured if: (i) conversion to sinus rhythm has not occurred until 1 h after the fourth dose, or (ii) the patient exhibits adverse or toxic effects (20). It has also been recommended to initiate QND sulfate treatment guided by the plasma QND concentrations; a plasma QND concentration of 2–5 μg/mL has been suggested based on therapeutic concentrations of 2–6 μg/mL of drug in human medicine (20, 22). This order of plasma concentration has been confirmed for use in horses (20), but has limited applicability for practitioners who cannot use therapeutic drug monitoring (TDM). In addition, despite the standardization of dosing regimens, a wide variability in plasma concentration has been observed for a given dosing regimen, as some cases may not achieve therapeutic concentrations while others reach toxic concentrations (7, 18). This is due to the inter-individual variability in plasma concentrations associated with a given dose, with each horse having its own pharmacokinetic parameters. These include plasma clearance and oral bioavailability, which determine the internal exposure when QND is administered orally. Nowadays, population pharmacokinetics (POP PK) using the non-linear mixed-effect model (NLMEM) is implemented to measure and explain with covariates [such as breed, age, sex, and health status the inter-individual variability in plasma concentration profiles in horses (23)]. Using a POP PK model, it is possible to explore and propose a rational empirical dosing regimen for oral QND in horses, that is, a dosing regimen that can be applied *a priori* to any horse without recourse to TDM. Indeed, using Monte Carlo simulations (MCS), a POP PK model can predict the plasma concentration profiles corresponding to different dosing regimens (dose and dosing intervals) to predict the probability of reaching either therapeutic or toxic concentrations in a given percentile of a horse population, that is, the probability of target attainment (PTA) of effective concentrations and concentrations generating adverse events (24). The PTA of efficacious and non-toxic plasma concentrations should typically be 90%.

This study aimed to demonstrate the variability in plasma QND concentrations between healthy horses and horses with AF. Our objective was to determine the range of therapeutic and toxic QND concentrations in horses, to propose an empirical dosing regimen that is effective, while also minimizing the risk of adverse drug effects for different PTA.

## 2 Materials and methods

### 2.1 Animals

Ten healthy Thoroughbred horses (2–7 years-old, five stallions and five mares, body weight [BW]: 473–563 kg) and 19 Thoroughbred racehorses that developed AF (2–10 years-old, 12 stallions and seven mares, BW: 430–540 kg) were used in this study. Date of onset of AF was unknown in 8 horses, and the average days from onset to start of the quinidine treatment was 4.6 ± 1.5 days in the remaining 11 horses. Horses with AF were hospitalized at two hospitals: Miho Training Center and Ritto Training Center, Japan. All horses had *ad libitum* access to water and hay. The study design was reviewed and approved by the Institutional Animal Care and Use Committee of the Equine Research Institute, Japan Racing Association (approval no. 22–8, 23–6). Written informed consent was obtained from the owners for participation of their horses in this study.

### 2.2 Administration of QND

For six healthy horses, a single dose PO and IV administration study was conducted with a two-week washout period. For single PO administration, 20 mg/kg QND sulfate dihydrate (molecular formula [MF]: C_40_H_48_N_4_O_4_ • H_2_SO_4_ • 2H_2_O, MW: 782.94 g/mol; 16.58 mg/kg as QND base, MF: C_20_H_24_N_2_O_2_, MW: 324.42 g/mol; Viatris Inc., Canonsburg, Pennsylvania, USA, Quinidine sulfate dihydrate VTRS powder for oral) was dissolved in 500 mL of water and administrated via nasogastric tube; the tube was flushed with 500 mL of water immediately after drug administration. For IV administration, 5 mg/kg QND hydrochloride monohydrate (MF: C_20_H_24_N_2_O_2_ • HCl • H_2_O, MW: 378.89 g/mol; 4.28 mg/kg as QND base; Merck KGaA Darmstadt, Germany) was dissolved in 500 mL of sterile saline and administrated into the right jugular vein as a slow 5-min infusion, using a 16G catheter that was inserted under the effect of local anesthesia (1 mL lidocaine) of the skin. Four healthy horses were administrated with 20 mg/kg QND sulfate dihydrate (16.6 mg/kg as QND base) q 6 h twice by the same method (as single PO administration). For the 19 AF horses, the dose and interval of drug administration were determined by the prescribing veterinarians, between 9.3 and 30.6 mg/kg QND sulfate dihydrate (7.7–25.4 mg/kg as QND base) and administrated via a nasogastric tube, and flushed similar to that as for the healthy horses. During treatment, surface electrocardiograms were recorded in all healthy horses and horses with AF (Nihon Koden, Tokyo, Japan, BMS2401).

### 2.3 Blood sampling

For IV administration group, blood samples were collected prior to drug administration and at 0 min (immediately after administration), 5, 10, 20, 30, 45 min, and 1, 2, 3, 4, 6, 8, and 12 h after IV administration. For a single PO administration, blood samples were collected prior to administration and at 30 min, 1, 1.5, 2, 2.5, 3, 4, 5, 7, 9, 12, and 24 h after administration. For the twice administration PO studies, blood samples were collected prior to administration and at 30 min, 1, 1.5, 2, 2.5, 3, 5.8 (before the second administration at 6 h), 6.5, 7, 7.5, 8, 8.5, 9, 12, and 24 h after the first administration. For horses with AF, blood samples were collected prior to and at 0.5, 1, 2, 3, and 4 h after administration, and the precise times of administration and sampling were recorded. Approximately 10 mL of the blood samples were collected in heparinized vacuum blood collection tubes from the right jugular vein using a 16G catheter, which was inserted under local anesthesia. The samples were immediately centrifuged at 1500 × *g* for 10 min, and the separated plasma samples were stored at −20°C until analysis.

### 2.4 Sample analysis

QND in the plasma was assayed using a liquid chromatography system (Shimadzu Corporation, Kyoto, Japan) connected to a mass spectrometer (SCIEX, Framingham, Massachusetts, USA). Control samples for the calibration of the plasma analysis were prepared by adding standard QND (Merck KGaA) to blank horse plasma. To 20 μL of plasma, 200 μL of acetonitrile and 20 μL of 1 μg/mL Quinidine-d3 (Toronto Research Chemicals Inc., Toronto, Canada) as internal standard were added. The sample was incubated for 5 min at 24°C and centrifuged at 10,000 × *g* for 5 min at the temperature of 24°C. Approximately 1 μL of each sample was injected into the liquid chromatography system connected to a mass spectrometer. Liquid chromatography separation was performed on the Acquity BEH (100 mm × 2.1 mm i.d., 1.7 μm) (Waters, Milford, Massachusetts, USA) with a mixture of 25 mmol/L ammonium formate,0.1% formic acid with the phrase and acetonitrile at a flow rate of 0.5 mL/min. The final calibration curve had a coefficient of correlation (*R*^2^) >0.995 over the concentration range of 0.03–10.0 μg/mL with a 1/y^2 weighing factor for QND. The lower limit of quantitation (LLOQ) was 0.03 μg/mL. Intra-day and inter-day accuracy and precision in quality control samples were determined at concentrations of 0.03, 0.1, 1, and 10 μg/mL (five replicates each). Accuracies were between 88.3 and 109.0%, and the precision of coefficient of variation (CV) was <15%.

### 2.5 Pharmacokinetic analysis

Plasma pharmacokinetic analysis was conducted using NLMEM with the commercially available software Phoenix WinNonlin and NLME (Certara, Version 8.4). The QND sulfate dihydrate for PO and QND hydrochloride monohydrate for IV dosage were expressed in terms of the QND base (conversion ratio of 1.206 for QND sulfate dihydrate and 1.168 for QND hydrochloride monohydrate). Quinidine plasma concentration values below the LOQ, that were encountered in less than 5% of the data, were excluded from the model (25, 26). A three-compartment structural model was selected based on the likelihood ratio test and the Akaike information criterion. The model was parameterized in terms of clearance and volumes of distribution. The estimated parameters were the central (V1) and peripheral (V2 and V3) volumes of distribution, plasma clearance (CL), and intercompartmental distribution clearances (CL2 and CL3). The absorption rate constant (Kabs) and the bioavailability factor (*F*) were added to the model for PO administration. A statistical model describing between-subject variability (BSV) was included in the population model. The inter-individual variability for a given parameter was described using an exponential model of the following form Equation 1:


(1) $$\theta_{\mathrm{parameter}\_i}=\theta_{tv\_\mathrm{parameter}}\;.\;EXP\left(\eta_{i}\right)$$

where *θ*_parameter_i_ is the value of θ for a given parameter in the i^th^ animal, θ_tv_parameter_ is the typical population value of the parameters, and *η_i_* is the deviation associated with the i^th^ animal from the corresponding θ population value. The distribution of *η* was assumed normal with a mean of 0 and a variance ω^2^. The inter-individual variability estimated with ω^2^, the variance term, was reported as the coefficient of variation (CV%), as follows Equation 2:


(2) $$CV\left(\%\right)=100\times \sqrt{\exp\left(\omega^{2}\right)-1}$$

Shrinkage of the random effects (*η*) toward the mean was described as Equation 3:


(3) $$\mathrm{shrinkage}=1-\frac{var\left(\eta_{r}\right)}{\omega^{2}}$$

where var(*η*_r_) is the variance of Empirical Bayes (“*post hoc*”) estimates (EBEs) of *η_s_*. When the shrinkage of *η* was >0.3, the data did not allow for a robust estimation of this random component. In this study, all the *η* shrinkage values were < 0.3. A full OMEGA matrix was used to determine the random components of the model, including the BSV associated with the fixed pharmacokinetic parameters.

The residual model was an additive, as well as a multiplicative (proportional) model of the form Equation 4.


(4) $$C\left(t\right)=f\left(\theta ,\mathrm{Time}\right)\times \left(1+\varepsilon_{1}\right)+\varepsilon_{2}$$

with ε_1_ is the multiplicative error term having a mean of 0 and a variance noted σ_1_ (Equation 5)


(5) $$\varepsilon 1\approx N\left(0,\sigma 1^{2}\right)$$

and ε_2_ is the additive error term having a mean of 0 and a variance noted σ_2_ (Equation 6)


(6) $$\varepsilon 2\approx N\left(0,\sigma 2^{2}\right)$$

The additive sigma was reported as its standard deviation noted with the same units as plasma concentration (μg/L), and the multiplicative sigma was reported as coefficient of variation. Moreover, covariates were tested for condition (healthy or AF), age, BW, and sex. The stepwise covariate search mode of Phoenix NLME was used to define statistically significant covariates for each structural parameter. The stepwise forward or backward addition or deletion of covariate effects (by adding one at a time) determined the improvement in the final model based on the Bayesian information criterion (BIC). A BIC value of 6.635 was used for adding a covariate and a value of 10.823 was used for deleting a covariate. As there was no model with BIC <10.0 compared to models without covariates, no covariates were included in the final model (25). A quasi-random parametric expectation–maximization (QRPEM) engine was used to maximize the likelihood.

MCS was used to generate plasma concentrations of a virtual population of 5,000 horses using individual predictions (IPRED) (*η* was as estimated), corresponding to the classical dosage regimen of 22 mg/kg QND sulfate (18.2 mg/kg as QND base, assuming that QND sulfate was the dihydrate compound; see Discussion) administered twice every 6 h PO administration (26). To propose a new effective dosage regimen, we simulated plasma QND concentrations for various regimens. The loading and maintenance doses required to achieve the targeted plasma QND concentrations were calculated using the following Equations 7 and 8 (27, 28):


(7) $$\mathrm{Loading}\;\mathrm{dose}=\frac{\mathrm{Target}\;\mathrm{concentration}\times \mathrm{tvVss}}{tvF}$$

(8) $$\mathrm{Maintenance}\;\mathrm{dose}=\frac{\mathrm{target}\;\mathrm{concentration}\times \mathrm{tvCl}}{tvF}$$

where tvCl is the typical plasma clearance value for a given dosing interval (here, 2 h), tv*F* is the typical bioavailability factor value, and tvVss is the typical steady-state volume of distribution. These typical values are listed in Table 1. In addition, dosing regimens were simulated to meet clinicians’ requests (gradual increase of doses) and take into account the constraints of clinical practice (in particular, administration interval) while optimizing and ensuring the safe and effective drug concentrations.

**Table 1:** Bootstrap estimates of typical (median) population primary and secondary parameters of quinidine in 27 horses.

Primary structural parameters	Units	Typical values (Median)	CV%	2.50%	97.50%	BSV%
V1	L/kg	0.63	12.4	0.49	0.80	40.5
V2	L/kg	0.59	12.1	0.49	0.72	37.8
V3	L/kg	3.68	7.0	3.09	4.13	28.5
CL	L/kg/h	0.49	6.4	0.43	0.56	25.6
CL2	L/kg/h	2.87	15.2	2.08	3.68	74.9
CL3	L/kg/h	2.44	13.9	1.91	3.22	47.3
Kabs	1/h	1.00	24.5	0.67	1.70	94.3
*F*	%	36.4	2.9	34.1	38.1	33.1
CMultStdev0 (residual, proportional for IV)	Scalar	0.0594	28.3	0.0058	0.0776	
CMultStdev1 (residual, proportional for PO)	Scalar	0.1571	7.8	0.1380	0.1853	
Stdev0 (residual, additive for IV)	μg/L	0.0338	31.6	0.0137	0.0547	
Stdev1 (residual, additive for PO)	μg/L	0.0479	47.5	0.0008	0.0923	
Secondary parameters						
Half_life_alpha	h	0.06	10.2	0.05	0.07	
Half_life_Beta	h	0.31	11.9	0.24	0.38	
Half_life_Gamma	h	7.76	8.8	6.40	8.68	
Absorption_Half_life	h	0.69	23.1	0.41	1.03	
Vss (steady-state volume of distribution)	L/kg	4.90	5.1	4.07	5.65	
MRT (Mean residence time)	h	10.12	9.3	8.30	11.41	

BSV%, CV%, 2.5, and 97.5% percentiles give the precision of the typical value estimates.

For F, an ilogit transformation was used to prevent estimates higher than 100%.

BSV, Between subject variability of structural parameters; V1, volume of distribution of the central compartment; V2, V3, volume of distribution of the peripheral compartments; CL, plasma clearance; CL2, CL3, distribution clearances; Kabs, absorption rate constant; F, bioavailability; CMultStdev, proportional component of residual error; stdev, additive component of the residual; tv, typical value; Vss, steady-state volume of distribution; MRT, mean residence time.

Two target plasma concentrations were explored in this study: 2.0 and 2.9 μg/mL. The first is the therapeutic concentration observed in this experiment and other similar studies (7, 20), while the second is the average value between the therapeutic concentration (2.0 μg/mL) and the concentration considered as unsafe in the present experiment (3.8 μg/mL).

### 2.6 Therapeutic and adverse effect concentration

In all healthy horses and horses with AF, the development of serious side effects, including depression, sweating, abnormal electrocardiogram, tachycardia >100 bpm, and colic, was recorded. In addition, the time to conversion to sinus rhythm was recorded for horses with AF. The QND plasma concentrations in the 60 min preceding the conversion of AF to sinus rhythm or those corresponding to the occurrence of adverse effects were considered therapeutic or toxic concentrations, respectively.

## 3 Results

**Figure 1:**
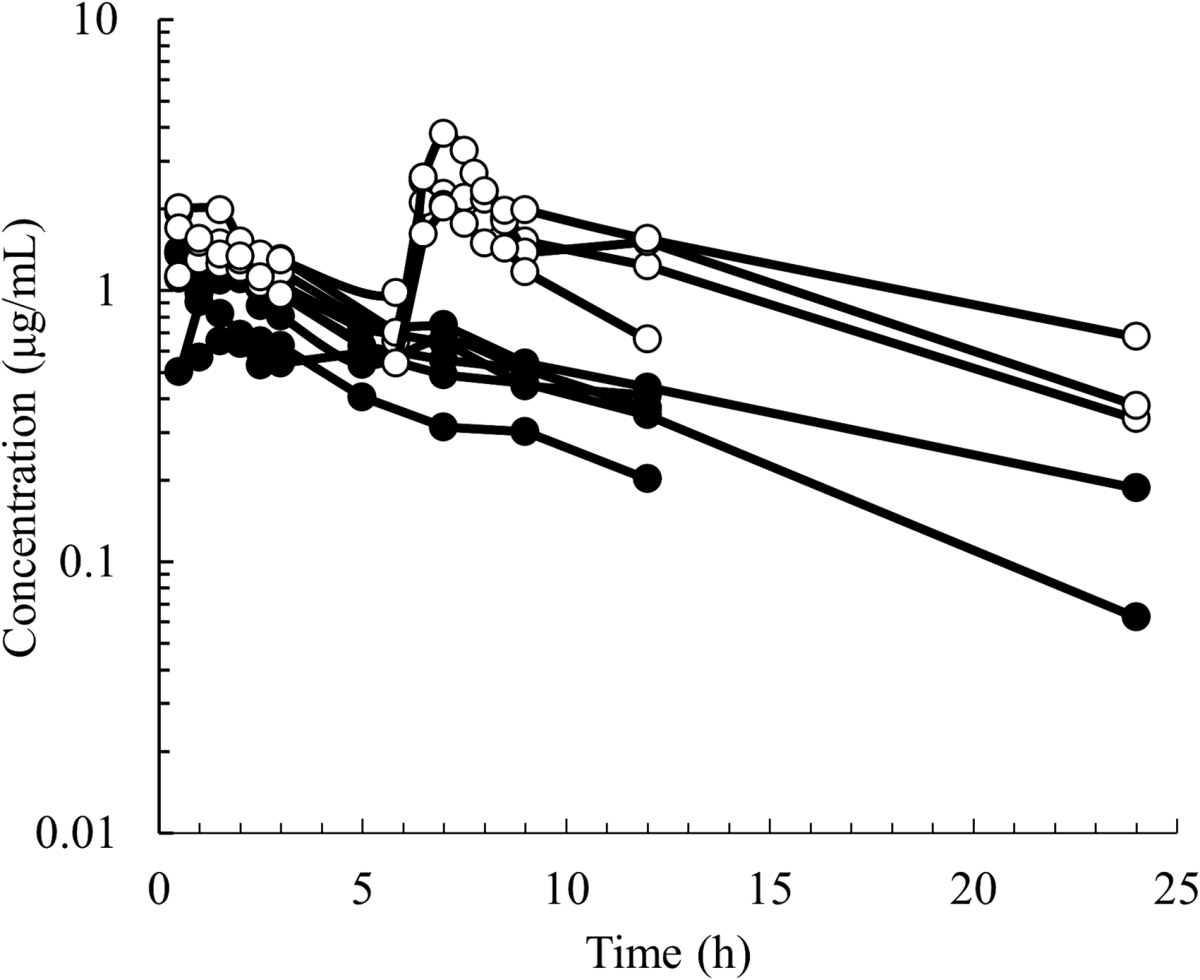
Semilogarithmic spaghetti plots of the disposition curves of quinidine after administration of QND sulfate dihydrate at 20 mg/kg BW (16.58 mg/kg as QND base) in 6 healthy horses (black circle) at a single PO dose and in 4 healthy horses (open circle) administered twice at 6 h intervals.

**Figure 2:**
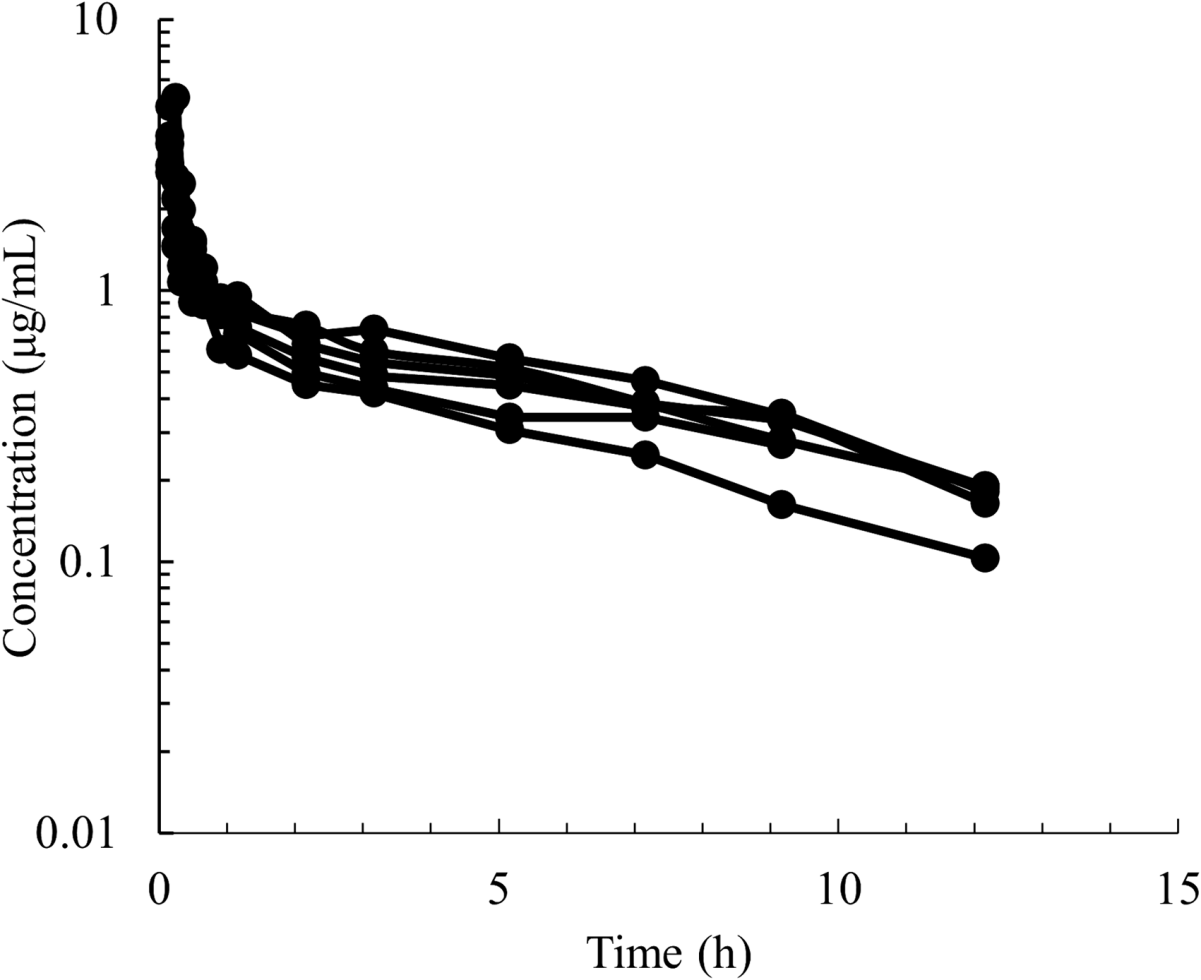
Semilogarithmic spaghetti plots of the disposition curves of quinidine after a single IV dose administration of 5 mg/kg BW of QND sulfate (4.28 mg/kg as QND base) in 6 healthy horses.

**Figure 3:**
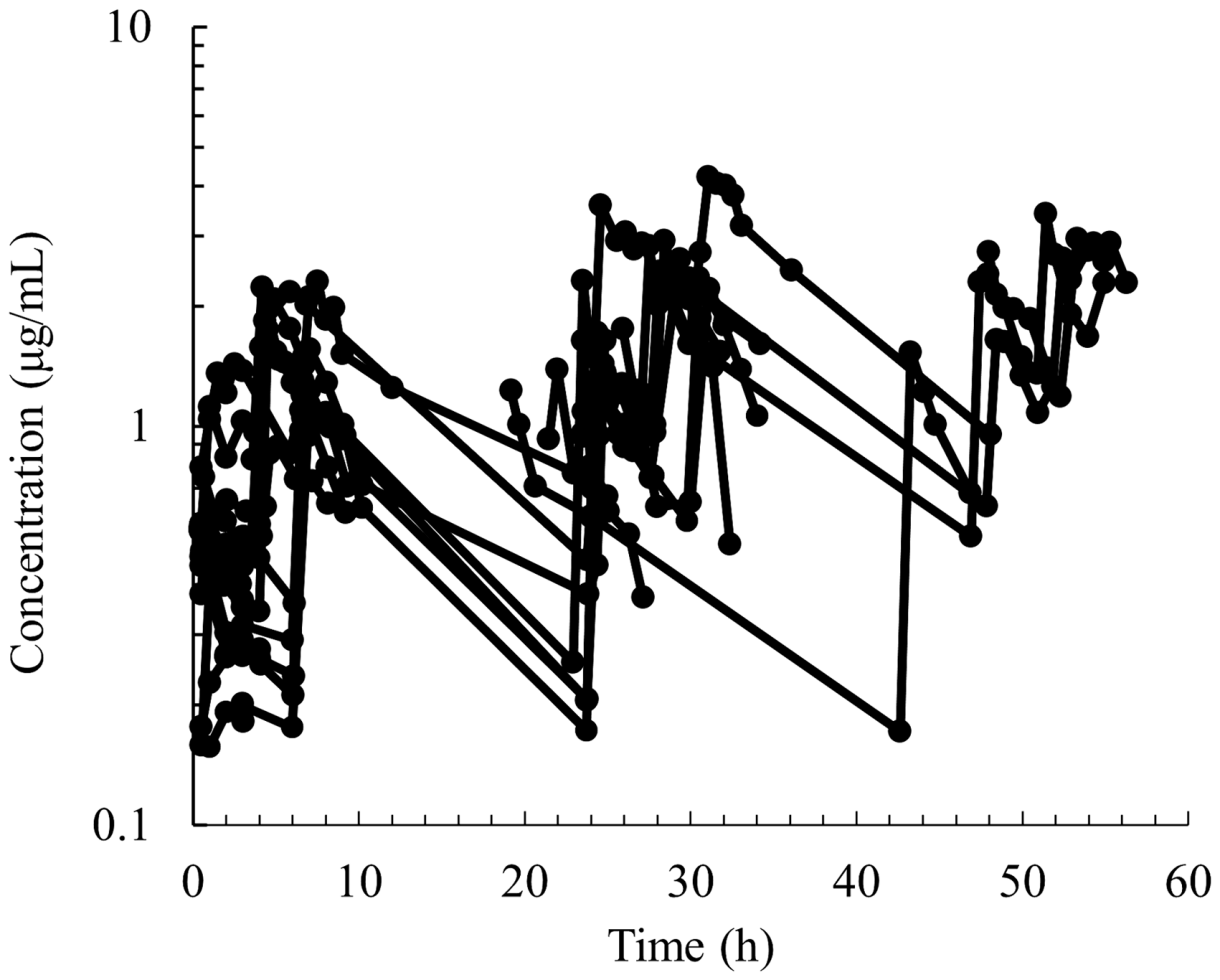
Semilogarithmic spaghetti plots of the disposition curves of quinidine after multiple PO dose administrations between 9.3–30.6 mg/kg of QND sulfate dihydrate (7.7–25.4 mg/kg as QND base) in 19 horses that developed atrial fibrillation.

**Figure 4:**
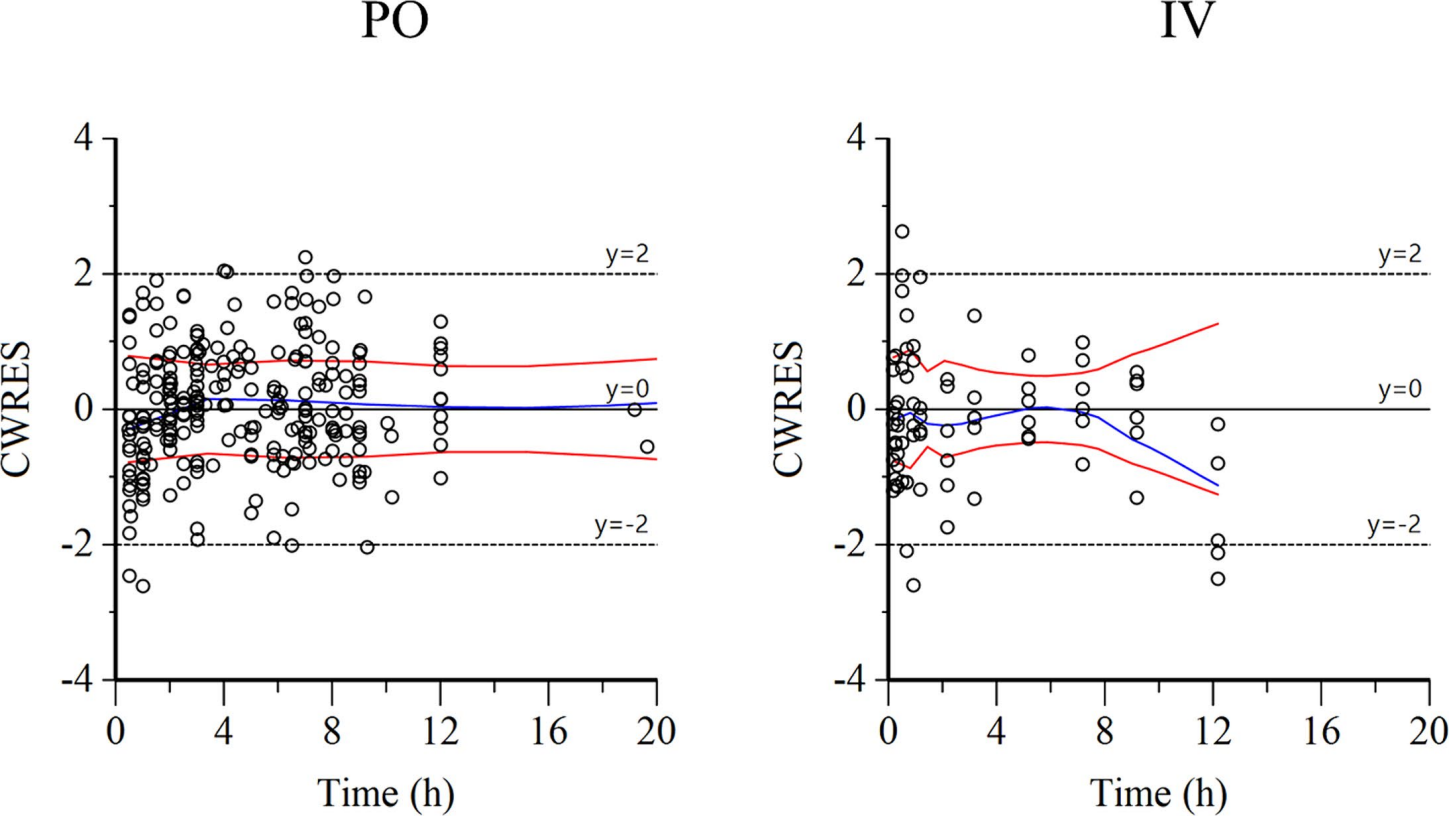
Conditional weighted residuals (CWRES) vs. time plot for PO administration (left) and IV administration (right). Values of CWRES were approximately N (0, 1) and hence concentrated between *y* = −2 and *y* = +2. Values significantly above 3 or below −3 should be suspected and may indicate a lack of fit and/or model misspecification. Inspection of the figure indicates that data were evenly distributed about zero and that the trends (as given by the blue line and the red line with its negative reflection) did not show any fanning, thus indicating no bias in the structural model.

**Figure 5:**
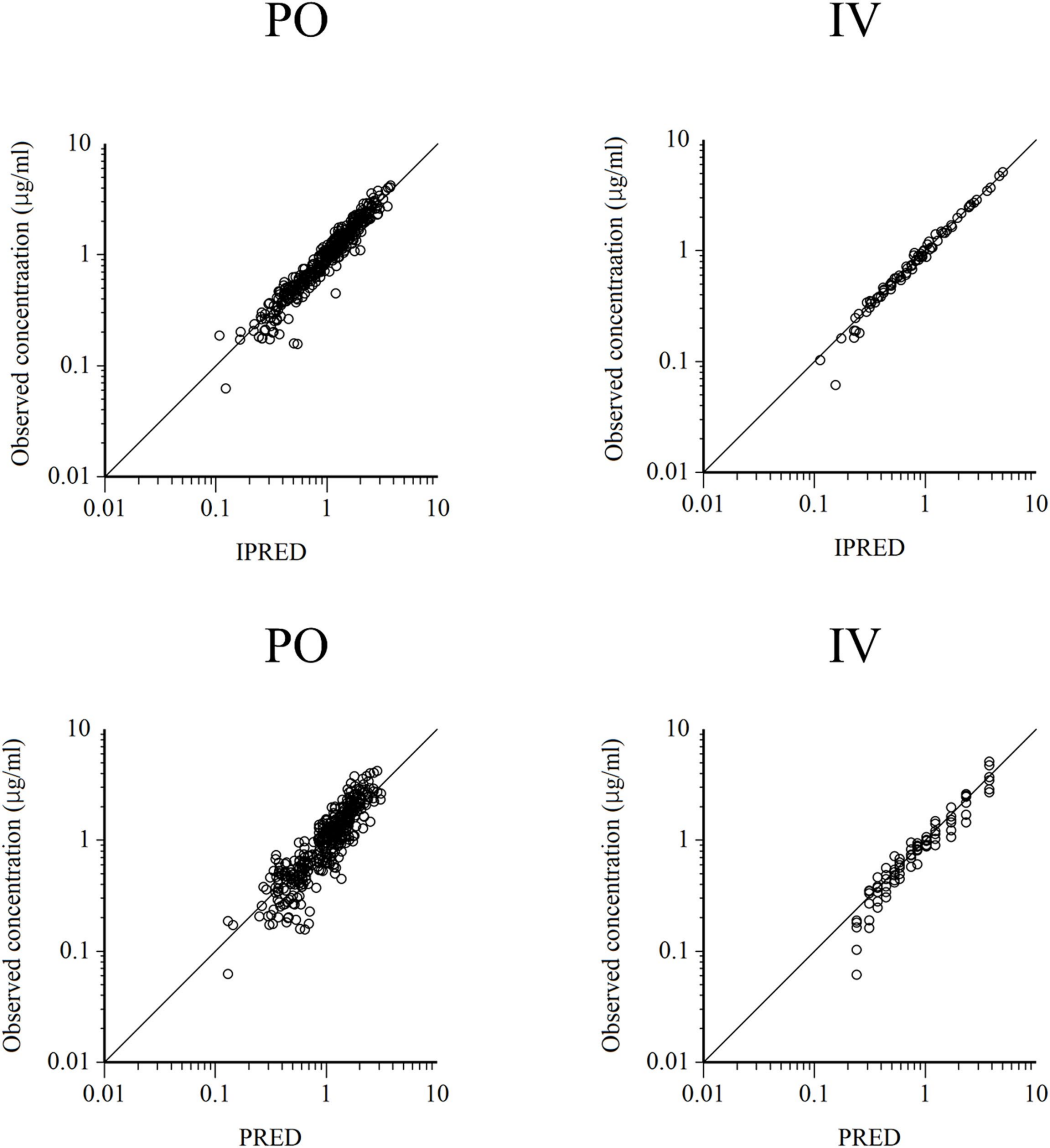
Logarithmic plots of observed quinidine plasma concentrations vs. individual predictions (IPRED) (upper) and population predictions (PRED) (bottom) after PO (left plots) and IV (right plots) administrations.

**Figure 6:**
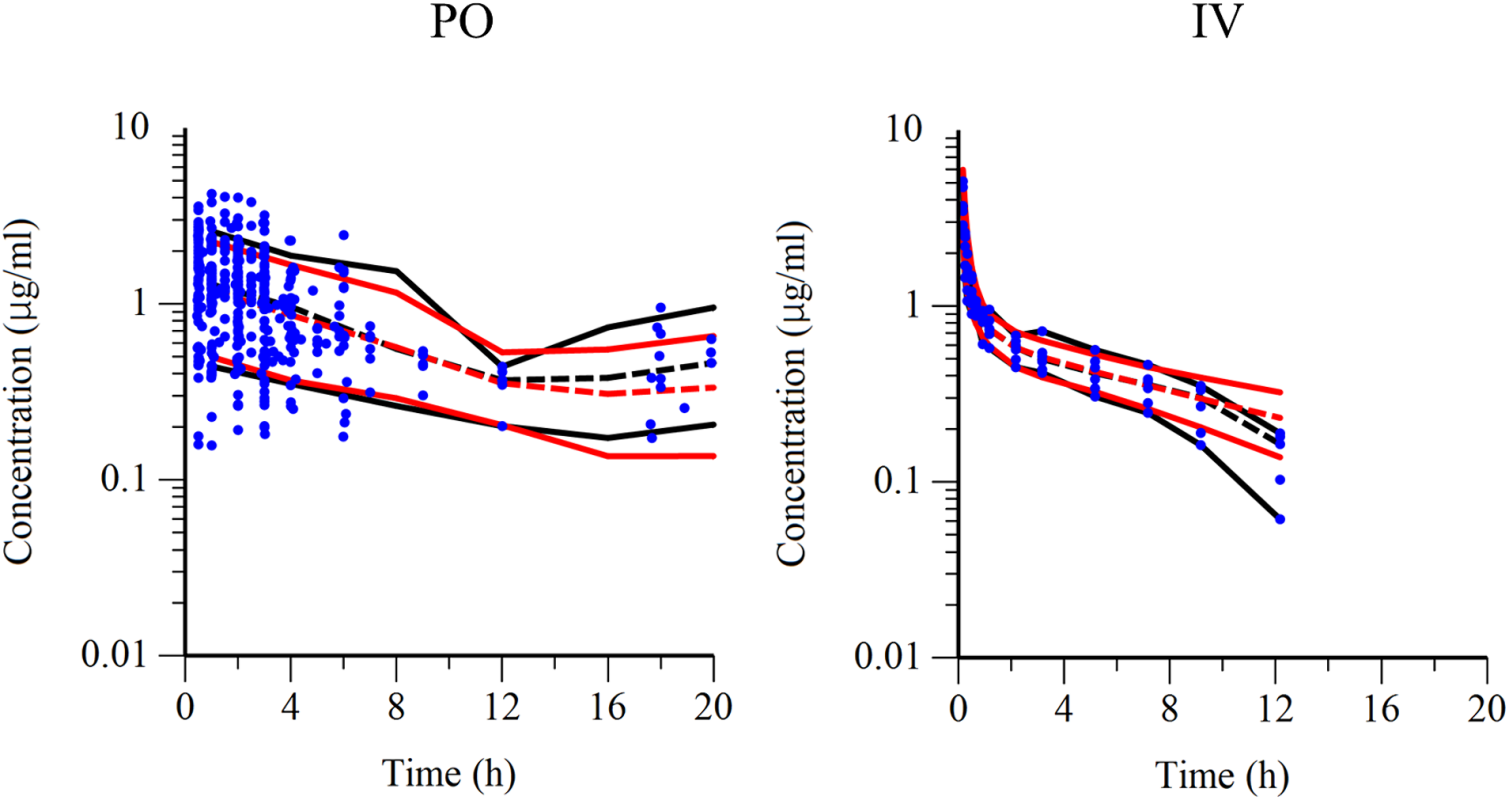
Visual predictive check of observations vs. time after PO administration (left) and IV administration (right). The observed and predicted 10th and 90th percentiles are indicated using solid black and red lines, respectively. The observed and predicted 50th percentiles (median) are indicated using black and red dotted lines, respectively. Blue dots represent individual raw data.

Semilogarithmic plots of the disposition curves of QND concentration in healthy horses are depicted in Figures 1, 2, and those for horses with AF are depicted in Figure 3. The plot of the conditional weighted residuals (CWRES) vs. time indicated that the residuals were randomly scattered around zero with no systematic trend, supporting the selection of the residual error model (Figure 4). Logarithmic plots of the observed drug plasma concentrations versus population prediction (PRED) and IPRED are shown in Figure 5. Data were evenly distributed around the line of identity, indicating no major bias in the population components of the model. Bootstrap estimates of the typical values of primary structural parameters of the model (*θ*), secondary parameters, and their associated coefficients of variation as a measure of the precision of their estimation are given in Table 1. A visual predictive check ensured that the simulated data from the final model were consistent with the observed data (Figure 6).

Of the 19 horses with AF, 18 horses showed conversions to sinus rhythm after QND sulfate administration, whereas one horse did not show any change in rhythm. For 13 horses, the conversion to sinus rhythm occurred during the blood sampling period, but for five other horses, the conversion occurred later. The median plasma QND concentration associated to sinus conversion in 13 horses was 2.0 μg/mL (range: 0.5–2.7 μg/mL). Adverse effect was observed in two healthy horses after IV administration and four AF horses after oral administration, and their median plasma concentration was 3.8 μg/mL (range: 1.6–5.1 μg/mL). Four horses developed depression, one developed sweating and colic, and one developed depression and tachycardia. Based on these results, a narrow range of therapeutic concentrations to be achieved with an appropriate dosage regimen was set between 2.0 and 3.8 μg/mL.

**Figure 7:**
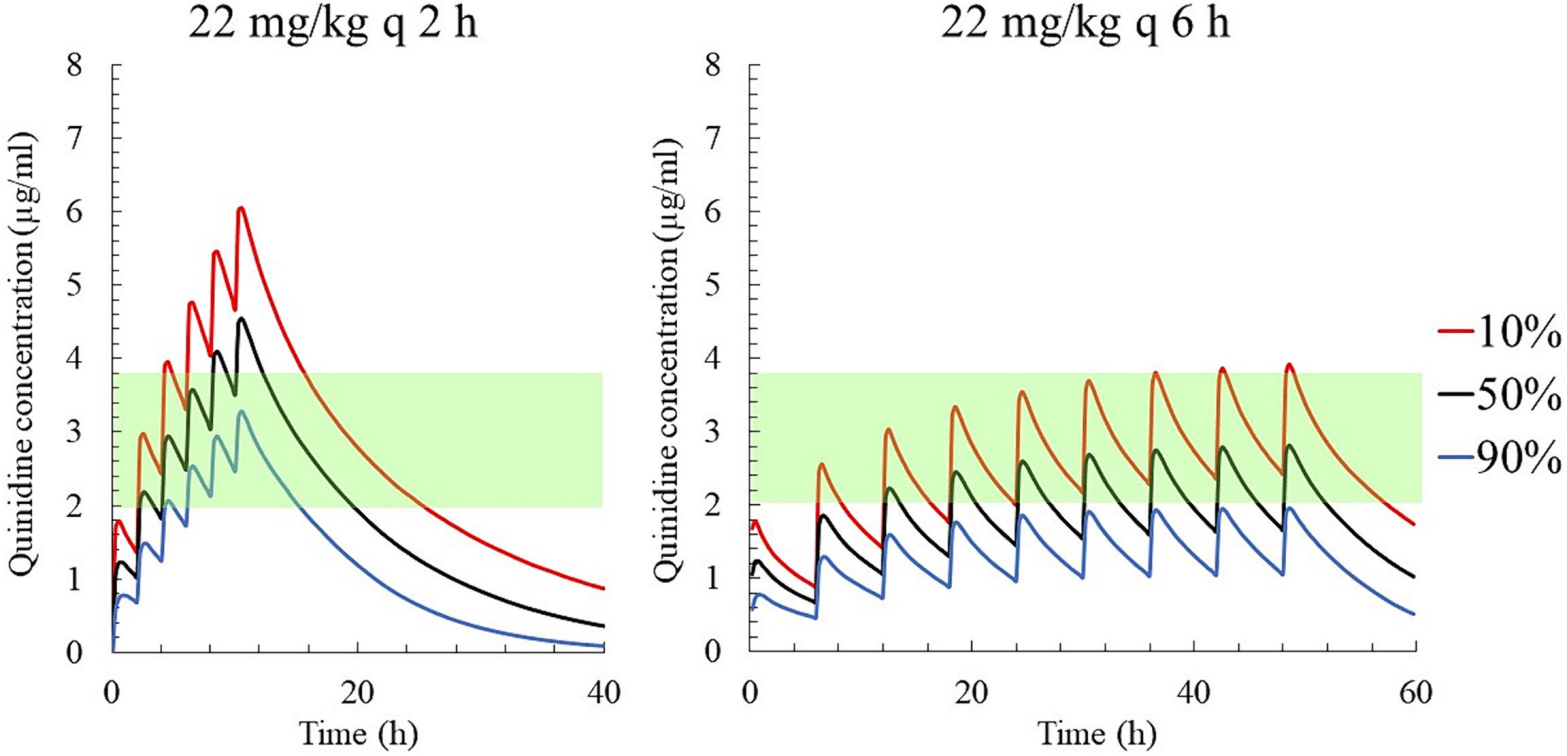
The predicted quinidine concentrations for 5,000 simulated horses based on Monte Carlo simulations after PO administration of quinidine sulfate dihydrate 22 mg/kg (18.24 mg/kg as QND base) q 2 h (left) and q 6 h (right). The predicted concentrations for 10, 50% (median), and 90% of the populations are indicated using red, black and blue lines, respectively. The green area indicates the therapeutic range (2.0–3.8 μg/mL).

The predicted QND concentration in the hypothetical 5,000 horses with AF, after PO administration of 22 mg/kg QND sulfate dehydrate (18.24 as QND base) q 2 h or q 6 h (the current recommended regimen) is shown in Figure 7. After the 4th q 2 h administration, 90% of the population reached QND concentration above 2.5 μg/mL, but 10% were above 4.8 μg/mL. After q 6 h administration, the 90 and 10% of the population reached QND concentration 1.9 and 3.9 μg/mL, respectively.

**Figure 8:**
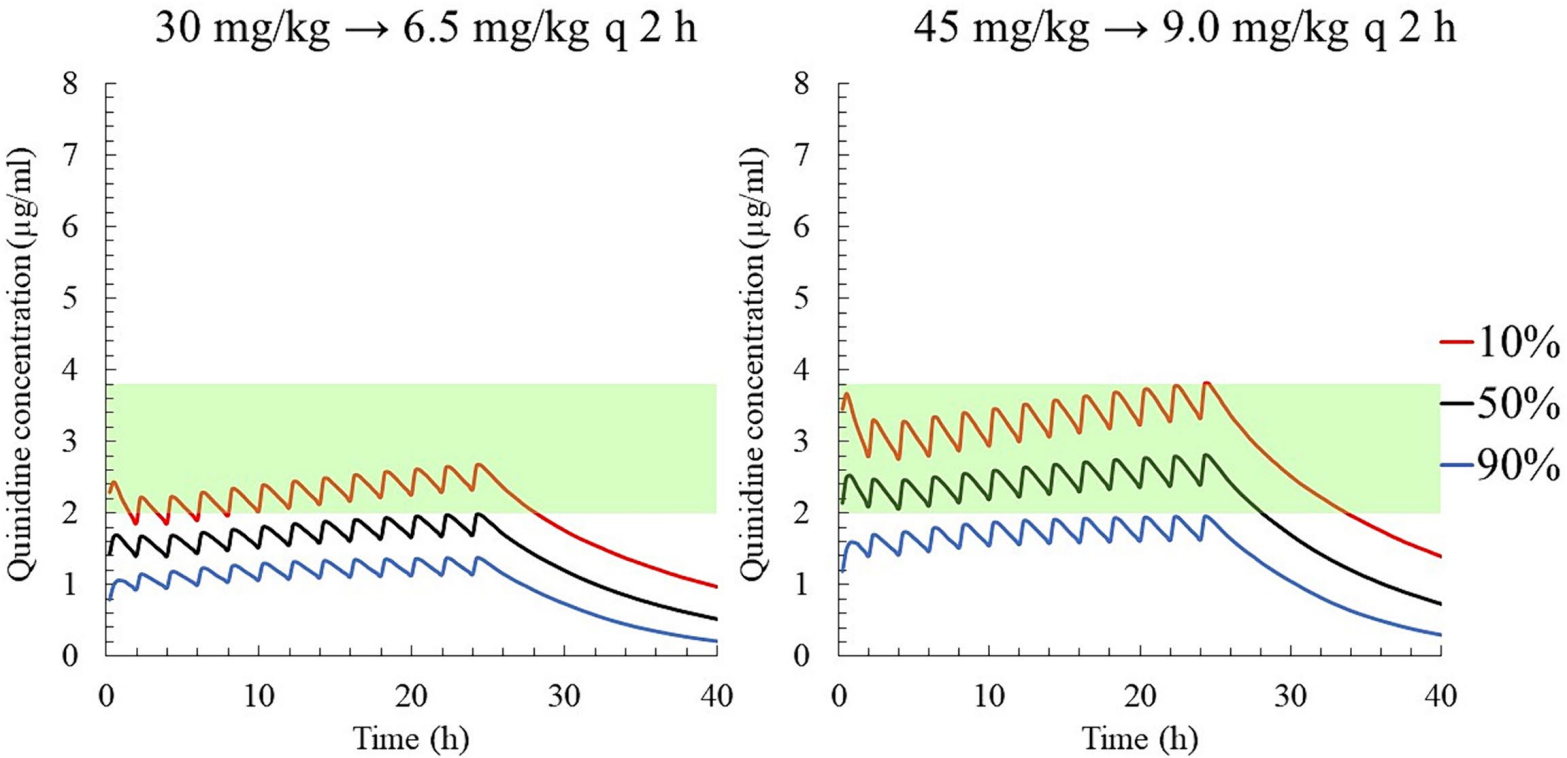
The predicted quinidine concentration for 5,000 simulated horses based on Monte Carlo simulations after PO administration of quinidine sulfate dihydrate 30 mg/kg (24.87 mg/kg as QND base) as loading dose and 6 mg/kg (4.97 mg/kg as QND base) q 2 h (left), and 45 mg/kg (37.31 mg/kg as QND base) as loading dose and 9 mg/kg (7.46 mg/kg as QND base) q 2 h (right). The predicted concentrations for 10, 50% (median), and 90% of the populations are indicated using red, black and blue lines, respectively. The green area indicates the therapeutic range (2.0–3.8 μg/mL).

When the target concentration was set at 2.0 μg/mL, which was the median therapeutic concentration in this study, the average loading and maintenance doses of QND sulfate dihydrate were calculated to 32.40 mg/kg (26.86 mg/kg as QND base) and 3.26 mg/kg/h (2.70 mg/kg as QND base), respectively (Table 2). In addition, 46.98 mg/kg QND sulfate dihydrate (38.95 mg/kg as QND base) and 4.73 mg/kg/h (3.92 mg/kg as QND base) were calculated for the target concentration of 2.9 μg/mL, which was the median value between therapeutic and toxic concentrations in this study. After calculating these initial values, dosing regimens that were easily prescribed by clinicians were simulated. QND sulfate dihydrate 30 mg/kg (24.87 mg/kg as QND base) as loading dose and 6.5 mg/kg (4.97 mg/kg as QND base) q 2 h as maintenance dose for 24 h was simulated, and QND plasma concentration at 24 h for 90, 50 and 10% of the horse population were found to be 1.4 μg/mL, 2.0 μg/mL, and 2.7 μg/mL, respectively (Figure 8). When the loading dose of QND sulfate dihydrate was increased to 45 mg/kg (37.31 mg/kg as QND base) and the maintenance dose to 9 mg/kg (7.46 mg/kg as QND base) q 2 h, the plasma QND concentrations in 90, 50, and 10% of the horse population were found to be 1.9 μg/mL, 2.8 μg/mL, and 3.8 μg/mL, respectively (Figure 8).

**Table 2:** Calculated loading dose and maintenance dose of quinidine (QND) sulfate dihydrate and QND base for each target concentration using typical values of parameters.

Target concentration (μg/mL)	Loading dose as QND base (mg/kg)	Loading dose as QND sulfate dihydrate (mg/kg)	Maintenance dose as QND base (mg/kg/h)	Maintenance dose as QND sulfate dihydrate (mg/kg/h)
0.5	6.72	8.10	0.68	0.82
1.0	13.43	16.20	1.35	1.63
1.5	20.15	24.30	2.03	2.45
2.0	26.86	32.40	2.70	3.26
2.5	33.58	40.50	3.38	4.08
2.9	38.95	46.98	3.92	4.73
3.5	47.01	56.70	4.73	5.71

**Figure 9:**
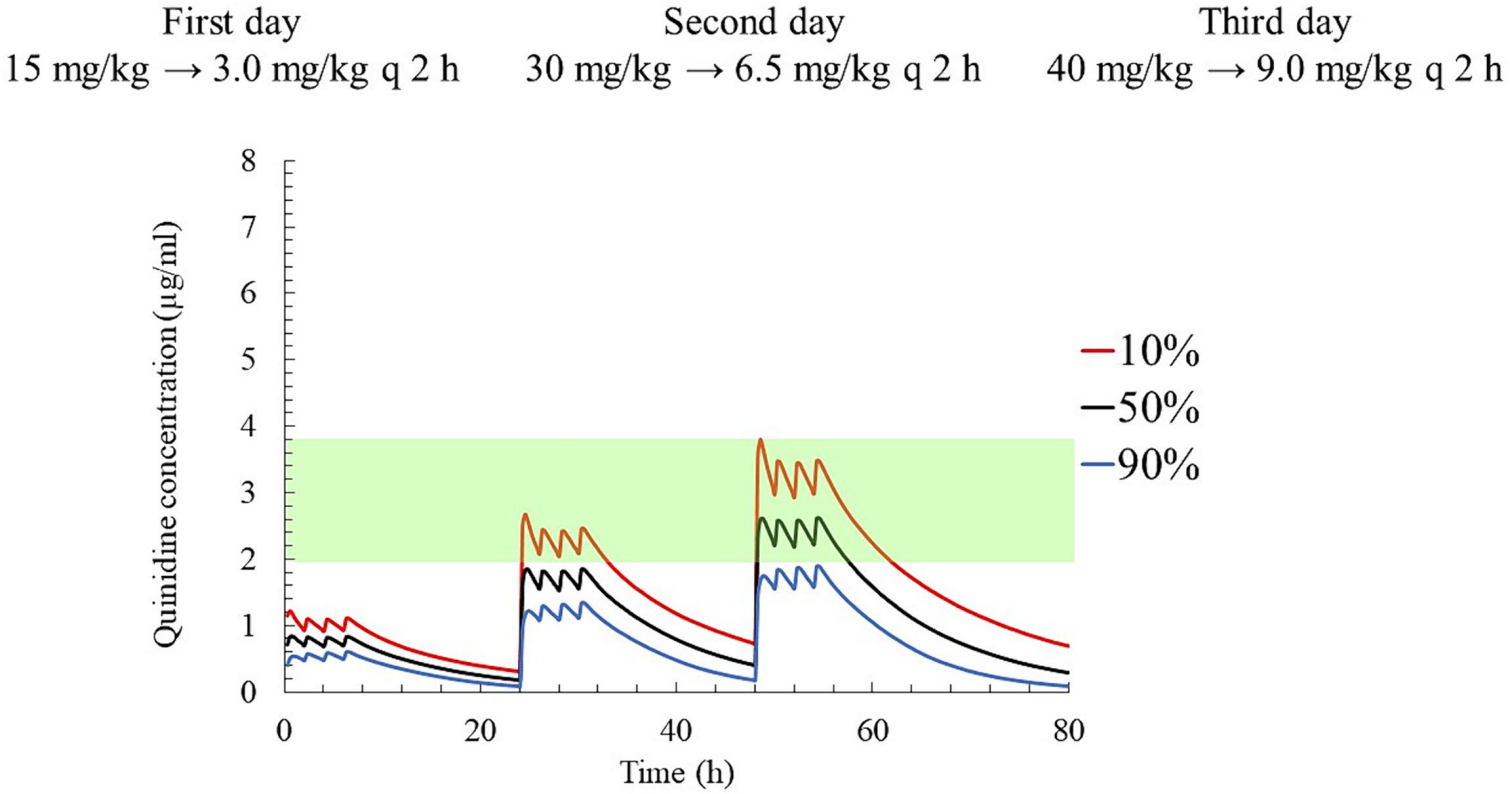
The predicted quinidine concentration for 5,000 simulated horses based on Monte Carlo simulations after administration of quinidine sulfate dihydrate 15 mg/kg (12.44 mg/kg as QND base) as loading dose and 3 mg/kg (2.49 mg/kg as QND base) q 2 h three times as maintenance dose on first day, 30 mg/kg (24.87 mg/kg as QND base) as loading dose and 6.5 mg/kg (4.97 mg/kg as QND base) q 2 h three times on second day, and 40 mg/kg (33.16 mg/kg as QND base) as loading dose and 9 mg/kg (7.46 mg/kg as QND base) q 2 h three times on third day. The predicted concentrations for 10, 50% (median), and 90% of the populations are indicated using red, black and blue lines, respectively. The green area indicates the therapeutic range (2.0–3.8 μg/mL).

Finally, dosing regimens characterized by progressive increases in the plasma concentrations of QND over 3 days were also simulated: on the first day, a loading dose of QND sulfate dihydrate was administered at 15 mg/kg (12.44 mg/kg as QND base); then, after a delay of 2 h, three maintenance doses of 3 mg/kg each (2.49 mg/kg as QND base) were administered every 2 h. On the second and third days, the loading doses were increased to 30 and 40 mg/kg (33.16 mg/kg as the QND base), and the maintenance doses to 6.5 and 9.0 mg/kg respectively, while maintaining the same administration intervals. The QND concentration in 90, 50, and 10% of horses reached 0.6, 0.8, and 1.1 μg/mL on the first day, 1.3, 1.8, and 2.7 μg/mL on the second day, and 1.9, 2.6, and 3.8 μg/mL on the third day, respectively (Figure 9).

## 4 Discussion

The pharmacokinetics of QND in horses have previously been reported and several dosage regimens have been proposed (19–21, 29). However, the response to QND treatment displays large inter-individual variability, which is explained by the variability in plasma QND concentrations (18, 20). The average C_max_ or typical value of the terminal half-life in this study was similar to that reported in previous studies (19–21, 29). Previously proposed dosing regimens were based on the mean plasma concentrations of QND; in contrast, the present population study estimated and considered the inter-individual variability of QND disposition in Thoroughbred horses to calculate empirical dosing regimens aimed at maximizing the therapeutic concentrations and/or minimizing the probability of undesirable effects in a given percentile of horses. The BSV of CL (25.6%) and that of *F* (33.1%) were relatively wide, explaining the large inter-individual variability in plasma exposure to QND. None of the covariates explored (age, BW, sex, and presence/absence of AF) were significant with BIC <10.0 compared to models without covariates, and the dosing regimen proposed in this study can probably be applied to the entire Thoroughbred population. The simulated dosages were expressed in QND sulfate dihydrate and their equivalent QND bases. In literature, dosage of QND sulfate is generally reported without specifying whether the drug was anhydrous or its sulfate dihydrate salt, which can be the possible cause of bias (of the order of 5%) between the expression of dosages in the present study and that in previous literature.

A classical recommended dosage regimen for horses for QND sulfate by nasogastric tubing is 22 mg/kg q 2 h until: (i) conversion to sinus rhythm, (ii) the appearance of adverse or toxic effects, or (iii) a total of four (to six) doses are administered. A fourth administration is only carried out if there are no adverse effects after the third administration; however, in this circumstance, it is recommended to measure the plasma concentration of QND after the 4th dose. From our Monte Carlo simulations, we estimated that a plasma QND concentration of 4.8 μg/mL was obtained in 10% of the population after the 4th administration of this classical dosage regimen. Although this concentration was within the therapeutic range (2–5 μg/mL) recommended by Reef et al. (20), it exceeded the median toxic concentration (3.8 μg/mL) that was established in the present study. We believe that this classical recommended dosage regimen should be re-evaluated, particularly because the maximum plasma QND concentration can quickly elevate. A safer alternative is a dose of 22 mg/kg QND sulfate every 6 h, but the duration which the lower 10% of the population are able to maintain the therapeutic concentration is shorter (26).

In an attempt to improve the current (but hazardous) recommendations and stably maintain QND therapeutic concentrations, we explored dosage regimens that were computed using typical values of estimated PK parameters and target QND concentrations of 2.0 or 2.9 μg/kg. When targeting QND concentration of 2.0 μg/mL, which was the observed median therapeutic concentration, 50% of the population was expected to reach this therapeutic value. When targeting QND concentration of 2.9 μg/mL, which was median value between median therapeutic and toxic concentrations, it was expected to include the largest portion of the population in the therapeutic range (a PTA of 80% was achieved between 2.0 and 3.8 μg/mL). However, variability in the response to QND concentration is a concern for these dosage regimens. Adverse effects were observed at 1.6 μg/mL for one horse, and there is a risk to use these simulated dosage regimens from the first administration. It is considered safer to gradually increase the plasma concentrations in horses. Therefore, other dosage regimens have been explored for clinical applications with the goal of fulfilling the practical expectations of clinicians.

Owing to the requirement for constant monitoring of serious adverse events associated with QND therapy (26), our hospitals aim to avoid late-night treatments when staffs are limited. Considering this, a dosage regimen was proposed in which the concentration was gradually increased over 3 days of daytime treatment. On the third day, the loading dose was adjusted from 45 mg/kg to 40 mg/kg avoiding plasma concentration higher than 3.8 μg/mL in at least 90% of the horses. On the first day, the dosage regimen achieved a concentration of approximately 1.0 μg/mL, which is a test dose level recommended in textbooks (26). By the second day, the plasma QND concentration of 50% of the population reached around 2.0 μg/mL, with 10% of population achieving a maximum plasma concentration of 2.7 μg/mL, ensuring an AF conversion to sinus rhythm in our cases. On the third day, the QND concentration reached a level where majority of the horses were within the therapeutic range and expected to convert to sinus rhythm, but was lower than the unsafe concentration of 3.8 μg/mL. At this stage, the clinician can choose whether to maintain or increase the QND concentration based on each horse’s individual response. Additionally, if frequent administration using a nasogastric tube is unacceptable to the horse, maintenance doses can be switched to oral administration using a syringe pump, dissolving the drug in 20 mL of water. However, after oral administration, plasma concentrations may be below the target concentration because of the possible loss of a part of the dose.

For horses, Reef et al. (20) recommended a range of plasma QND concentrations of 2–5 μg/mL based on therapeutic concentrations in human medicine (2–6 μg/mL) (20, 22). Most horses with plasma quinidine concentrations >5 μg/mL exhibited an adverse or toxic effect of QND (clinical or electrocardiographic) (20). In addition, studies have also reported that plasma QND concentration > 3 μg/mL is considered unsafe; therefore, 1.5–3 μg/mL concentration was proposed as the therapeutic range in horses (7). In the present study, we established 2.0–3.8 μg/mL as the new therapeutic window based on the median concentrations, ensuring AF conversion to sinus rhythm and minimization of adverse effects. In our experiment, the administered dose was increased with caution using 4–6 h dosing intervals, which is in contrast to a previous study by Reef et al. (20) that used a classical dosage regimen of q 2 h administration. Due to this difference in dosage regimen, a plasma QND concentration above 4 μg/mL was limited in this study, and most horses with AF were successfully converted to sinus rhythm with QND plasma concentration < 3 μg/mL. Analysis of the PK/PD relationship corresponding to this therapeutic range is lacking in both humans and horses, and further research is required to refine the estimation of our proposed therapeutic window. Correlations have been reported between surface ECG and sinus rhythm conversion or QND side effects in horses (20, 30). ECG analysis of AF cases in this study is also expected to determine the precise therapeutic window through PK/PD analysis.

In summary, this population study confirmed the large variability in QND plasma concentrations, which can be attributed to both the variability of plasma clearance and individual bioavailability. In addition, we simulated different dosing scenarios, including those recommended in previous literature, and proposed a dosing regimen that ensures that the largest population (PTA of 80%) would be able to reach the therapeutic window. However, it is difficult to propose an empirical dosing regimen capable of completely separating the range of therapeutic concentrations from those associated with risk of serious adverse effects (target PTA of 90%). This indicates that QND treatment in horses is a candidate for TDM. In this respect, our population study is an essential first step that needs to be completed to ensure that the intra-individual variability of QND disposition, unlike the inter-individual variability, is limited (in practice, less than 30%) to make such a plasma monitoring approach clinically useful. However, we must acknowledge there is also the problem of limited access to measurement equipment (e.g., LC/MS/MS) in veterinary clinics. To promote optimal TDM methods in equine hospitals, it will be necessary to determine the number of samples required for TDM, optimal collection times and evaluate the predictive value of this approach.

## Data Availability

The original contributions presented in the study are included in the article/supplementary material, further inquiries can be directed to the corresponding author.
